# Production of Bioactive Compounds by Actinomycetes and Their Antioxidant Properties

**DOI:** 10.1155/2014/217030

**Published:** 2014-03-26

**Authors:** Avilala Janardhan, Arthala Praveen Kumar, Buddolla Viswanath, D. V. R. Saigopal, Golla Narasimha

**Affiliations:** Department of Virology, Sri Venkateswara University, Tirupati, Andhra Pradesh 517502, India

## Abstract

An actinomycete was isolated from mangrove soil collected from Nellore region of Andhra Pradesh, India, and screened for its ability to produce bioactive compounds. The cultural, morphological, and biochemical characters and 16S rRNA sequencing suggest that the isolated strain is *Nocardiopsis alba*. The bioactive compounds produced by this strain were purified by column chromatography. The *in vitro* antioxidant capacity of the isolated compounds (fractions) was estimated and fraction F2 showed very near values to the standard ascorbic acid. The potential fraction obtained by column chromatography was subjected to HPLC for further purification, then this purified fraction F2 was examined by FTIR, NMR, and mass spectroscopy to elucidate its chemical structure. By spectral data, the structure of the isolated compound was predicted as “(Z)-1-((1-hydroxypenta-2,4-dien-1-yl)oxy)anthracene-9,10-dione.”

## 1. Introduction

Because of their useful biological activities, microbial secondary metabolites have received considerable attention especially in the beneficial effects of human health. Biosynthesis of these secondary metabolites through metabolic engineering and industrial biotechnology offers significant advantage over conventional methods for extraction from biomass. Among the microorganisms, marine bacteria produce unique and novel secondary metabolites and these organisms display interesting biological activities. Along with the different types of marine bacteria, actinomycetes also play an extensive role in the pharmaceutical and medical industry for their capacity to produce secondary metabolites with diverse chemical structures and biological activities. Thousands of bioactive compounds have been isolated and characterized, many of which have been developed into drugs for treatment of wide range of diseases in human, veterinary, and agriculture sectors [1–3]. Hence, the actinomycetes are considered to be the most potent source for the production of secondary metabolites, antibiotics, and other bioactive compounds. It is well established that each actinomycete strain has probably genetic potential ability to produce 10–20 secondary metabolites [4, 5]. A large body of evidences stated that actinomycetes are noteworthy as antibiotic producers, making 75% of all known products, and the* Streptomyces* has special role in antibiotic production [6, 7].* Streptomyces* yielded many therapeutic agents which include antibacterial such as tetracyclines, antifungal such as amphotericin, and anticancer drugs exemplified by Adriamycin and the immunosuppressant tacrolimus [8].* Streptomyces *has been reported to contribute nearly 70% of metabolites described under actinobacteria [9].* Streptomycetes* and related actinomycetes continue to be useful sources of novel secondary metabolites with a range of biological activities that may ultimately find applications as anti-infectives, anticancer agents, or other pharmaceutically useful compounds [10].

Therefore, screening, isolation, and characterization of promising strains of actinomycetes producing potential antibiotics and other therapeutics have been a major part of research [11, 12]. Recent studies are focusing on the response of antioxidant system of bacteria, which is important in terms of biotechnology, such as* Streptomyces* growth in various oxidative stress conditions [13].* Nocardiopsis *species is one of the actinomycetes which may produce different types of pharmacological compounds with antioxidant, antitumor, anti-inflammatory, antibacterial, and antioxidant properties. Searching for unique actinomycete that metabolized an essential component in natural product-based drug is becoming more and more interesting and meaningful. Antioxidants play an important role in inhibiting and scavenging free radicals, thus providing protection to humans against various infections and degenerative diseases [14]. Either increased free radicals or decreased antioxidant can lead to oxidative stress, which signifies the identification of natural antioxidative agents. There are certain naturally occurring antioxidants that can give protection against oxidative stress induced damage in human cells. Modern research is now directed towards natural antioxidants from plants and microorganisms which serves as safe therapeutics [15]. Therefore, the main objective of this work was the production and characterization of novel bioactive compound from marine actinomycetes and to screen their antioxidant properties.

## 2. Materials and Methods

### 2.1. Collection of Soil

Mangrove soil was collected from a field in Kandaleru creek, near Konamala, Gudur, Nellore (dist.), Andhra Pradesh, India. The soil sample was collected from 2 inches below the soil surface and soil samples were transferred to lab in sterilized polythene bags.

### 2.2. Isolation of Actinomycetes from Mangrove Soil

Starch casein agar (SCA) medium with 50% seawater was used for isolation of actinomycetes as described by El-Nakeeb [16] and Küster and Williams [17].

### 2.3. Screening for Bioactive Compound Producing Actinomycetes

The isolated strains were screened for the production of bioactive compounds. The isolated actinomycetes strains were inoculated in ISP2 medium with 50% seawater. The inoculated flask was kept for incubation at room temperature for a period of 5 days on rotary shaker (120 rpm) at 28°C. After incubation the broth was filtered and the filtrate was used to test antimicrobial activity. The overnight cultures like* E. coli* (ATCC 9837),* Staphylococcus aureus* (ATCC 6538),* Bacillus subtilis *(ATCC 9856),* and Pseudomonas aeruginosa* (ATCC 9027) were used as test organisms. The test organisms were spread uniformly on agar plate and wells were bored on the agar surface; then the wells were filled with the culture filtrate. Then plates were incubated at 37°C for 24 to 48 hrs.

### 2.4. Morphological and Taxonomical Identification of Isolated Strain

Based on the results of screening, one potential strain was selected for further investigations. The potential isolate (GN2) was observed for aerial spore, mycelia color, spore chain morphology, and other microscopic characters. The taxonomic identification of actinomycetes sp. was based on Nonomura's key and Bergey's Manual [18, 19]. Finally the strain was identified by 16s DNA sequencing.

### 2.5. Production and Extraction of Bioactive Compounds

All the media used in this study were prepared in 50 mL filtered seawater and 50 mL distilled water. Hence the growth was optimum at that proportion. Spores of potential actinomycete strain were scrapped from starch casein agar and inoculated into 50 mL of inoculation medium in 250 mL conical flask and kept in rotary shaker at 120 rpm for 48 hours at 28°C. Then 10% of inoculum was transferred into 100 mL of production medium and kept in rotary shaker at 120 rpm for 7 days at 28°C. After fermentation, mycelium and supernatant were separated first by filtration and finally by centrifugation at 10,000 rpm for 30 minutes at 4°C. The extracellular compounds from culture supernatant were extracted by liquid-liquid extraction method using equal amount of ethyl acetate and concentrated by Rota evaporation.

The crude extract (2 g) was subjected to silica gel column (15 × 2.5 cm with 200 to 300 mesh size) using different solvent systems. The separation of the crude extract was conducted via gradient elution with hexane: ethyl acetate. The solvent fractions F1, F2, F3, and F4 were collected at the concentration of 8 : 2 ratio; the purity of the compound was found by TLC at 2 : 8 ratio of mobile phase (hexane : ethyl acetate).

### 2.6. Determination of Biological Activities of the Bioactive Compounds

#### 2.6.1. Determination of the Total Phenolics

The total phenolic content of four fractions (bioactive compounds) was determined spectrophotometrically with Folin-Ciocalteu reagent, using a slightly modified method by Junaid et al. [20]. The extract was mixed with Folin-Ciocalteu reagent (1 : 1) and 4 mL of sodium carbonate (1 M) was added and allowed to stand for 15 minutes. The absorbance was read spectrophotometrically at 765 nm. A standard curve was plotted using different concentrations of gallic acid (standard, 0–1000 *μ*g/mL) and the total phenolic content of extract was estimated as *μ*g gallic acid equivalents (GAE)/mg of extract. The reaction was conducted in triplicate, and the results were averaged.

#### 2.6.2. Total Antioxidant Activity

Total antioxidant activity of the fractions was determined according to the method of Prieto et al. [21]. 0.3 mL of each fraction was mixed with 3.0 mL of reagent solution (0.6 M sulphuric acid, 28 mM sodium phosphate, and 4 mM ammonium molybdate). Reaction mixture was incubated at 95°C for 90 min in a water bath. Absorbance of all the sample mixtures was measured at 695 nm. Ascorbic acid (100 *μ*g/mL) was used as standard control.

#### 2.6.3. Qualitative Test for Free Radical Scavenging Activity

10–15 *μ*L of each fraction was spotted on the baseline of the silica gel plates (Himedia) as a spot for chromatographic separation and identification of the fractions using methanol : chloroform (95 : 5, v/v) as mobile phase. It was allowed to develop the chromatogram for 30 minutes. After completion of the chromatogram the whole plate was sprayed with 0.15% (w/v) DPPH solution using an atomizer [22].

#### 2.6.4. Quantitative Test for Free Radical Scavenging Activity by DPPH

Free radical scavenging activity of each fraction was assayed by DPPH (1,1-diphenyl-2-picrylhydrazyl) [23]. 2 mL of DPPH solution (0.002% in methanol) was mixed with 2 mL of different concentrations (5–200 *μ*g/mL) of each fraction and standard (ascorbic acid) in separate tubes. The tubes were incubated in dark at room temperature for 30 minutes and the optical density was measured at 517 nm using UV-Vis spectrophotometer. The absorbance of the DPPH control (without extract/standard) was noted. The scavenging activity was calculated using the formula

Scavenging activity (%) = [(*A* − *B*)/*A*] × 100, where *A* is absorbance of DPPH control and *B* is absorbance of DPPH in the presence of extract/standard.

#### 2.6.5. Total Reducing Power

Total reducing capacity of the extracts was determined according to the method of Subramaniam [24]. The bioactive compound containing fractions (100 *μ*g/mL) were mixed with 1% potassium ferricyanide and the mixture was incubated at 50°C for 20 min; 2.5 mL of 10% trichloroacetic acid (TCA) was added to the mixture and centrifuged at 5000 rpm for 10 min. The upper layer of solution (2.5 mL) was mixed with 2.5 mL of distilled water and 0.5 mL of 0.1% ferric chloride and the color developed was measured at 700 nm. Ascorbic acid (100 *μ*g/mL) was used as standard control.

### 2.7. Structure Elucidation

Among the four fractions F2 was more potent based on preliminary screening, that is, antioxidant capacity, so the purity of the F2 was confirmed by HPLC by using hexane and ethyl acetate as mobile phase at a flow rate of 1 mL min^−1^; detection was carried out by UV detector with 209 nm. Then the pure compound F2 structure was predicted by spectral analysis. All solvents used for spectroscopic and other physical studies were reagent grade and were further purified by standard methods [25]. Melting points (mp) were determined using a calibrated thermometer by Guna Digital MeltingPoint apparatus and expressed in degrees centigrade (°C). Infrared spectra (IR) were obtained on a Perkin-Elmer Model 281-B spectrophotometer. Samples were analyzed as potassium bromide (KBr) disks. Absorption was reported in wavenumbers (cm^−1^). ^1^H and ^13^C NMR spectra were recorded as solutions in DMSO-*d*_6_ on a Bruker AMX 400 MHz spectrometer operating at 400 MHz for ^1^H and 100 MHz for ^13^C. The ^1^H and ^13^C chemical shifts were expressed in parts per million (ppm) with reference to tetramethylsilane (TMS). LCMS mass spectra were recorded on a Jeol SX 102 DA/600 mass spectrometer.

### 2.8. Statistical Analysis

Experimental results are the mean ± standard deviation (SD). Statistical comparisons using one way analysis of variance (ANOVA) followed by Duncan's test for comparison of results between lichens samples and standard antioxidant with *P* < 0.05 was regarded as significance and *P* < 0.01 as very significant.

## 3. Results and Discussion

### 3.1. Isolation and Screening of Actinomycetes

**Figure 1:**
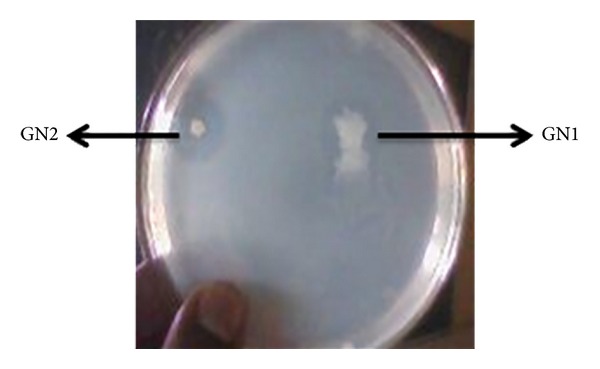
Isolated colonies of actinomycetes showing clear zone on SCA medium.

Two morphologically different strains along with clear zones around the colony were observed in the starch casein medium after 5 days of incubation (Figure 1). These two strains, GN1 and GN2, were screened for the production of bioactive compounds; both the strains exhibited good antimicrobial activity against different pathogenic bacteria as shown in Table 1. But the second strain which showed maximum activity was selected for morphological, cultural, molecular characterization and bioactive compound production. These results were correlated with the bioactive compounds from* N. pseudobrasiliensis *and* N. mediterranei* [26, 27].

### 3.2. Morphological and Biochemical Test

**Figure 2:**
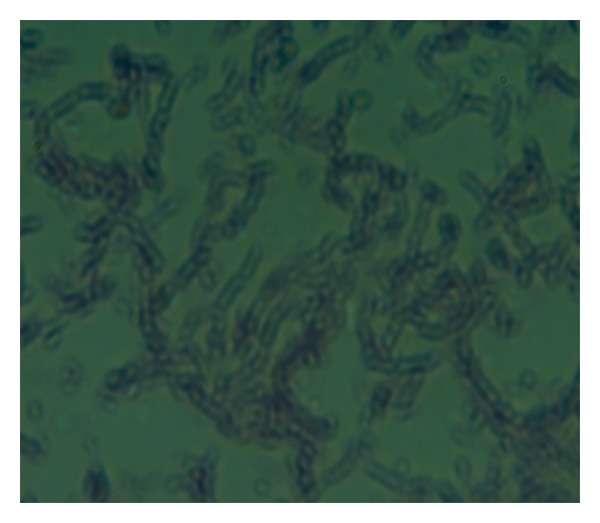
Microscopic picture of isolated strain.

Micro- and macroscopic characteristics: the aerial mycelium formed monopodially branched spore-bearing hyphae with the shape of loops, open or compact spirals with 3–6 curves (Figure 2). The strain was clearly polymorph and the colonies were completely covered by aerial mycelium and it formed a clear zone around the colony (Figure 1). These morphological characters are closely agreed with the findings of Goodfellow [28] and Hoshino et al. [29]. In carbon assimilation test the isolate GN2 grew poorly in the presence of sucrose and fructose as a sole carbon source and formed abundant mycelium on the media with glucose and maltose, whereas in nitrogen utilization test, it has grown abundantly on glutamic acid and poorly on histidine, methionine, and leucine. It also showed other biochemical characters like starch, casein, and gelatin hydrolysis as shown in Table 2.

### 3.3. Identification of Actinomycetes

**Figure 3:**
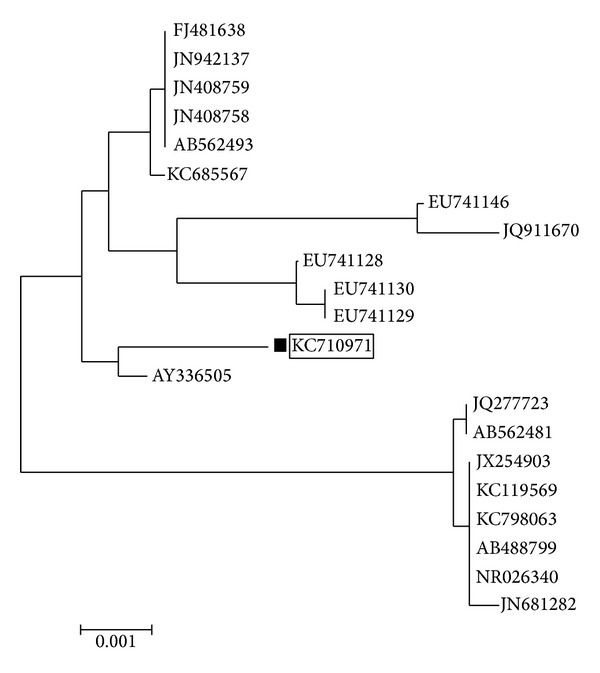
Phylogenetic tree of isolated actinomycetes GN2.

The taxonomic identification of the GN2 was based on 16s rDNA analysis. The 16s rDNA sequence of the strain was compared with the sequences in GenBank using BLAST and aligned with the sequences retrieved from NCBI GenBank database using the Clustal *W* method. The phylogenetic tree was constructed based on neighbor joining tree method and illustrated in Figure 3. The database was deposited in NCBI GenBank with an accession number KC710971. Based on the cultural, morphological, physiological, and molecular analysis, the GN2 was identified as* Nocardiopsis alba*.

### 3.4. Determination of Biological Activities of the Bioactive Compounds

#### 3.4.1. Determination of Total Phenolics and Total Antioxidants

**Figure 4:**
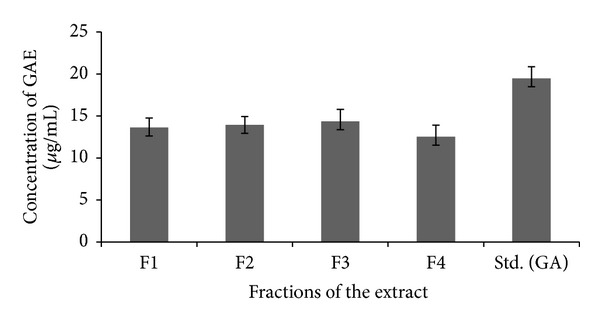
Total phenolics in the fractions from GN2 strain compared with standard gallic acid.

The antioxidant activities of bioactive compounds are mainly due to their redox properties, which can play an important role in absorbing and neutralizing free radicals [30]. The phenolic content of each of the fractions was estimated as 13.62 ± 1.12; 13.94 ± 0.98; 12.52 ± 1.39; and 14.37 ± 1.47 mgGA/g (F1, F2, F3, and F4), respectively, which is very similar to that of phenolic activity of the standard (ascorbic acid) (19.48 ± 1.37 mgGA/g) shown in Figure 4. The highest antioxidant capacity of bioactive compound could be attributed to the presence of high total polyphenol contents, since a positive correlation between phenolic composition and antioxidant activity was proved by Katalinic et al. [31]. The presence of the phenolic groups in the secondary metabolites is considered to be a key element for the antioxidative efficiency [32].

**Figure 5:**
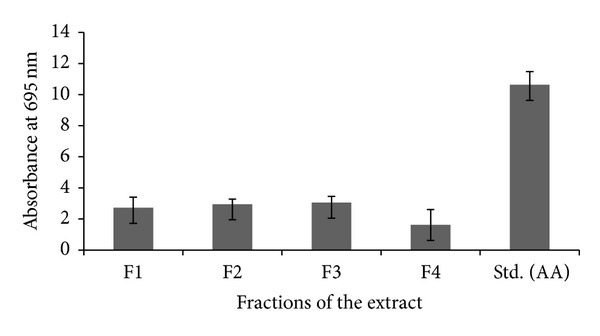
Total antioxidant activities of the fractions and standard antioxidant ascorbic acid.

**Figure 6:**
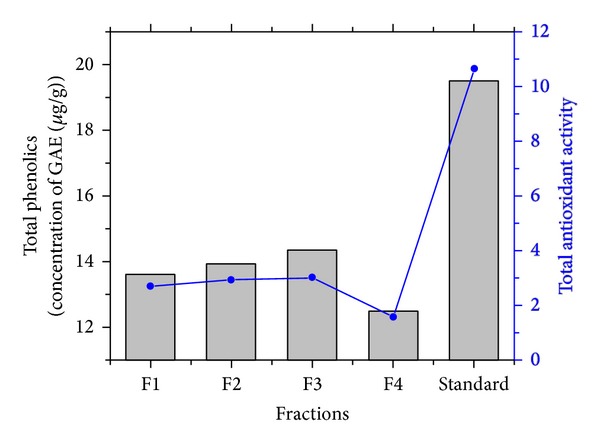
Variation of antioxidant capacity as a function of total phenolics content.

The bioactive compounds extracted from fractions showed very potent total antioxidant capacity. The results of experimental samples and the standard antioxidant (ascorbic acid) equivalents are presented in Figure 5. The results showed that the total antioxidant capacity of extracted samples was 2.72 ± 0.4, 2.95 ± 1.18, 3.05 ± 0.98, and 1.62 ± 0.4 AA/g, respectively, and the standard antioxidant ascorbic acid shows 10.63 ± 0.85 AA/g antioxidant activity. In the present experiment, total antioxidant activity was increased if total phenolics in terms of gallic acid equivalents were increased (Figure 6)

#### 3.4.2. Qualitative and Quantitative Test for Free Radical Scavenging Activity by DPPH

**Figure 7:**
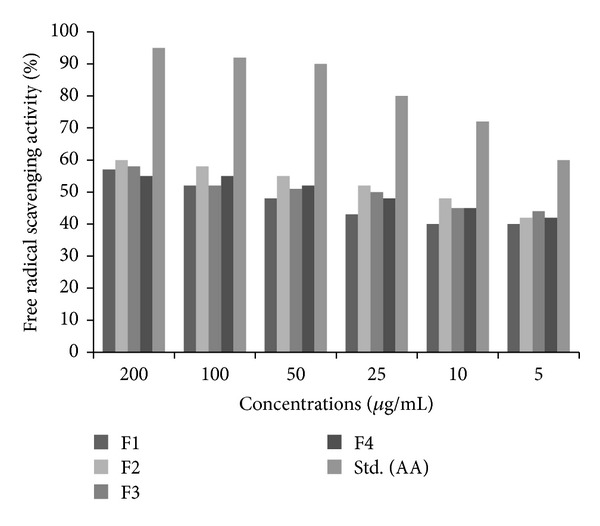
Free radical scavenging activities of four fractions and ascorbic acid.

The DPPH assay is one of the most common and relatively quick methods used for testing radical scavenging activity of biological active particles [33]. The chromatogram the plate was sprayed with DPPH (0.15% *W*/*V*) solution. The yellow color spots (Figure 7) indicated the presence of antioxidant nature of collected fractions with *R*_*f*_ values of 15, 12, and 10 cm. The results of radical scavenging effect of four fractions and ascorbic acid have exhibited dependent scavenging activity of DPPH radicals. Though the fractions were able to scavenge DPPH* (free radical) and convert it into DPPHH, the scavenging effect of the fractions was lesser than that of ascorbic acid. The radical scavenging effect of F2, F3, and F4 was greater than 50% at concentration of 50 *μ*g/mL and that fraction 1 was greater than 50% at concentration of 100 *μ*g/mL.

#### 3.4.3. Reducing Power

**Figure 8:**
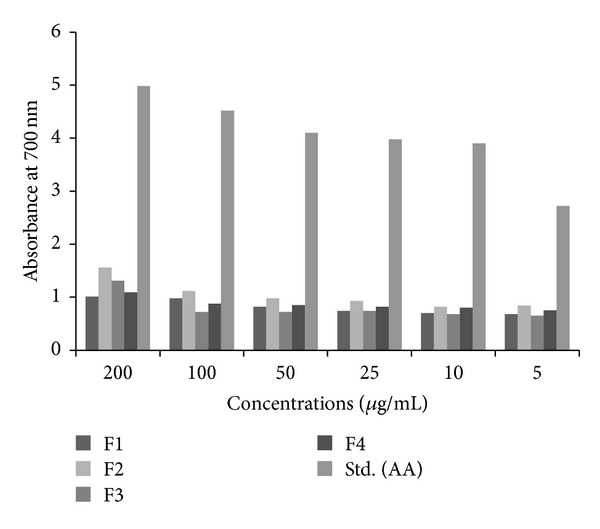
Ferric reducing activities of four fractions and ascorbic acid.

The reducing power of the four fractions was determined by the reduction of Fe^3+^ to Fe^2+^ in the presence of different concentrations of each fraction and ascorbic acid. The absorbance of reaction mixture at 700 nm increased with the increase in concentration of extract indicating reducing potential of extract. Measured values of absorbance varied from 0.68 to 1.56. Among the tested fractions, fraction 2 gave the highest reducing power, although the reducing activity was lower than the standard ascorbic acid as shown in Figure 8. The reducing capacity of the tested fractions decreased in the following order: fractions 2, 3, and 4 and fraction 1, respectively.

### 3.5. Structure Elucidation

**Figure 9:**
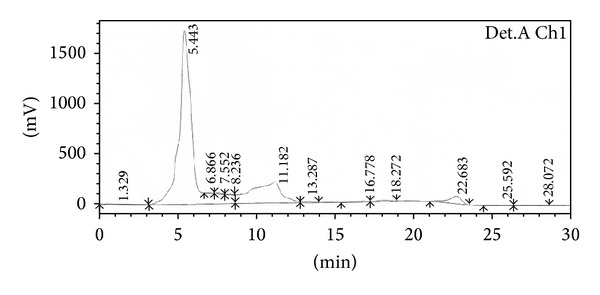
HPLC of isolated bioactive compound (fraction 2).

**Figure 10:**
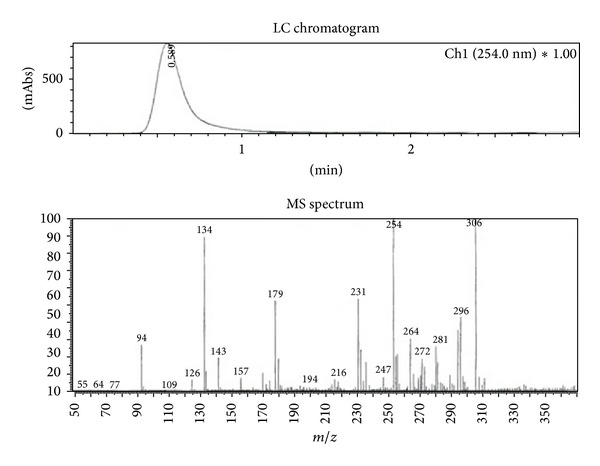
Mass spectrum of the F2 fraction.

**Figure 11:**
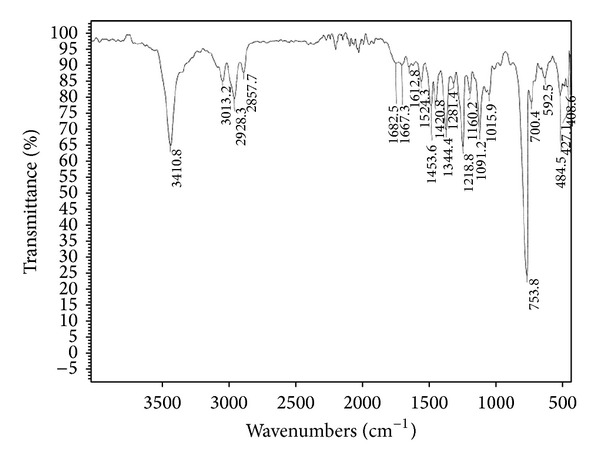
IR spectrum of the F2 fraction.

**Figure 12:**
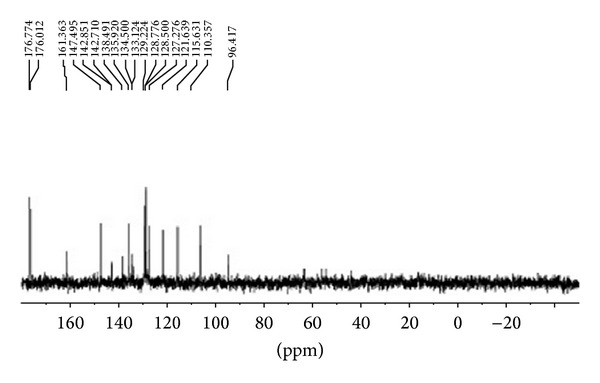
^13^C NMR spectrum of the F2 fraction.

The isolated pure compound showed a well developed peak having a retention time at 5 min (Figure 9). The molecular weight of this compound (F2) was determined by a mass spectrum and was found to be *m*/*z* 306.09 as a molecular ion (Figure 10). By the elemental analysis, the molecular formula of fraction 2 was identified as C_19_H_14_O_4_. Based on the molecular formula (C_19_H_14_O_4_) the double bond equivalents (DBE) value of fraction 2 was calculated as thirteen. After assuming a (DBE) value the two IR signal at signals observed at 1682 and 1667 cm^−1^ indicates the presence of two C=O groups indicates two double bonds equivalency. IR signal at 1612 cm^−1^ represents the conjugated C=C bond represents two double bonds equivalency. The IR signal at 3410 cm^−1^ represents the presence of aliphatic OH group. IR signal at 3013 cm^−1^ represents the presence of aromatic protons. Two signals at 2928 cm^−1^ and 2857 cm^−1^ and one signal at 1612 cm^−1^ represent the presence of conjugated =C–H signal (Figure 11). It was further supported by the presence of twelve aromatic carbons, two carbonyl carbons, and four conjugated double bonded carbons in its ^13^C NMR spectrum of sample. ^13^C signals at *δ* 176.7 and 176.0 are supporting to the two carbonyl carbons (Figure 12).

**Figure 13:**
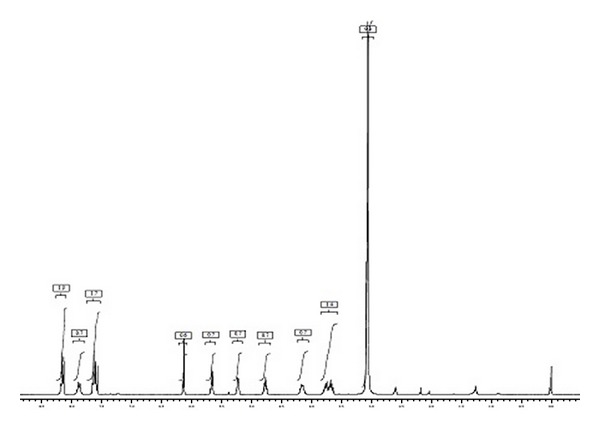
^1^H NMR of the F2 fraction.

**Figure 14:**
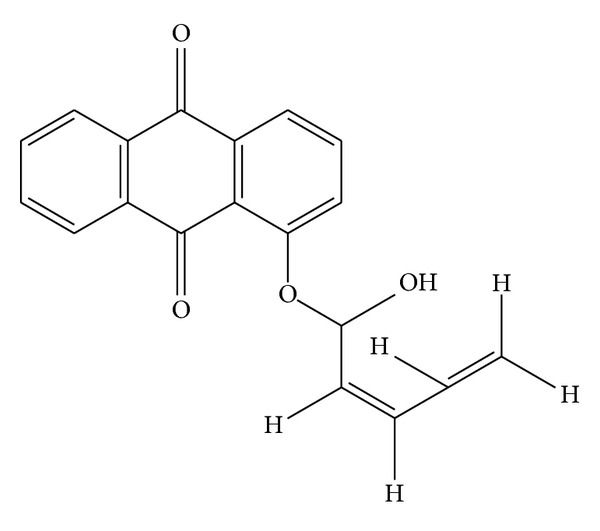
Structure of F2 fraction.

In ^1^H NMR the proton signals between *δ* 7.54 and 8.22 indicate the presence of seven aromatic protons. ^1^H NMR singlet signal at *δ* 5.23 represents the presence of aliphatic OH which is attached to ethereal carbon. The remaining ^1^H NMR signals between *δ* 3.11 and *δ* 6.2 characterize the aliphatic protons on the resonated carbons (Figure 13). By all the above observations the structure of the F2 is predicted and the name of compound is noted as “(Z)-1-((1-hydroxypenta-2,4-dien-1-yl)oxy)anthracene-9,10-dione” (Figure 14).

### 3.6. Spectral Data

Melting point (MP): 174–176°C; infrared (IR) (KBr): $\overset{-}{v}$ 1682 and 1667 (C=O), 2928 cm^−1^ and 2857 (=C–H), 1612 (C=C), 3410 (O–H), 3013 (ArC–H) cm^−1^; Proton NMR (^1^H NMR (400 MHz, DMSO-*d*_6_): d 8.12–8.22 (3H, m), 7.84–7.92 (^1^H, d), 7.54–7.67 (3H, m), 6.12–6.17 (1H, d), 5.61–5.7 (1H, d), 5.23 (1H, s, OH), 4.72–4.81 (1H, m), 4.1–4.21 (1H, m), 3.32 3.11 (2H, m). Carbon NMR (^13^C NMR) (100 MHz, DMSO-*d*_6_): d 176.7, 176.1, 161.3, 147.4, 142.8, 138.4, 135.9, 134.5, 133.7, 129.2, 128.7, 127.2, 121.6, 115.6, 110.3, 96.4. LC MS (%): *m*/*z* 306 (100%) [MH^+•^]; elemental analysis. C_19_H_14_O_4_.

## 4. Conclusion

Marine environments are particularly complex and have varied group of life forms, which occur in environments with extreme variations in pressure, salinity, and temperature. Owing to this nature, marine microorganisms have developed exceptional metabolic and physiological capabilities to be able to survive in such intense habitats that led them to produce different kind of metabolites, which could not be produced by the terrestrial microorganisms. Extensive research on marine natural products over the past three decades has revealed that marine actinomycetes are most prolific sources of novel and diverse metabolites. In the present study, an actinomycete,* N. alba*, was isolated from mangrove soil and screened for its ability to produce bioactive compound. The bioactive compounds produced by this strain were purified by column chromatography. The potential fraction obtained by column chromatography was subjected to FTIR, NMR, and mass spectroscopy to elucidate its chemical structure and the structure of the compound was predicted as “(Z)-1-((1-hydroxypenta-2,4-dien-1-yl)oxy)anthracene-9,10-dione”. The extracted bioactive compound has shown good antioxidant properties and further research is in progress to produce bioactive compound in large quantities and to make the compound an industrially important one.

## Figures and Tables

**Table 1:** Screening of actinomycetes by antimicrobial activity.

S. number	Test organisms	Zone of inhibition in cm	
		GN1	GN2
1	*E. coli *	0.9	1.5
2	*Staphylococcus aureus *	0.7	1.2
3	*Bacillus subtilis *	0.7	1.0
4	*Pseudomonas aeruginosa *	0.6	1.0

**Table 2:** Macroscopic and microscopic tests of GN2 strain.

Characters	GN2
Colony appearance	Mycelial (cottony)
Sporulation of aerial mycelia	Long chains
Motility	Nonmotile
Colony colour	White
Gram's staining	+
Starch hydrolysis	+
Gelatin hydrolysis	+
Casein hydrolysis	+

Carbon utilization	
Glucose	++
Sucrose	+
Fructose	+
Maltose	++

Nitrogen utilization	
Glutamic acid	++
Histidine	+
Methionine	+
Leucine	+
